# MR-Based Assessment of Bone Marrow Fat in Osteoporosis, Diabetes, and Obesity

**DOI:** 10.3389/fendo.2016.00074

**Published:** 2016-06-27

**Authors:** Christian Cordes, Thomas Baum, Michael Dieckmeyer, Stefan Ruschke, Maximilian N. Diefenbach, Hans Hauner, Jan S. Kirschke, Dimitrios C. Karampinos

**Affiliations:** ^1^Department of Diagnostic and Interventional Radiology, Klinikum rechts der Isar, Technische Universität München, Munich, Germany; ^2^Else Kröner Fresenius Center for Nutritional Medicine, Klinikum rechts der Isar, Technische Universität München, Munich, Germany; ^3^Section of Diagnostic and Interventional Neuroradiology, Klinikum rechts der Isar, Technische Universität München, Munich, Germany

**Keywords:** bone marrow, magnetic resonance imaging, magnetic resonance spectroscopy, osteoporosis, diabetes, obesity

## Abstract

Bone consists of the mineralized component (i.e., cortex and trabeculae) and the non-mineralized component (i.e., bone marrow). Most of the routine clinical bone imaging uses X-ray-based techniques and focuses on the mineralized component. However, bone marrow adiposity has been also shown to have a strong linkage with bone health. Specifically, multiple previous studies have demonstrated a negative association between bone marrow fat fraction (BMFF) and bone mineral density. Magnetic resonance imaging (MRI) and magnetic resonance spectroscopy (MRS) are ideal imaging techniques for non-invasively investigating the properties of bone marrow fat. In the present work, we first review the most important MRI and MRS methods for assessing properties of bone marrow fat, including methodologies for measuring BMFF and bone marrow fatty acid composition parameters. Previous MRI and MRS studies measuring BMFF and fat unsaturation in the context of osteoporosis are then reviewed. Finally, previous studies investigating the relationship between bone marrow fat, other fat depots, and bone health in patients with obesity and type 2 diabetes are presented. In summary, MRI and MRS are powerful non-invasive techniques for measuring properties of bone marrow fat in osteoporosis, obesity, and type 2 diabetes and can assist in future studies investigating the pathophysiology of bone changes in the above clinical scenarios.

## Introduction

Bone consists of the mineralized (i.e., cortex and trabeculae) and the non-mineralized component (i.e., bone marrow). The interaction of the mineralized and non-mineralized components plays an important role in bone loss pathophysiology. Quantitative measurements of the mineralized component have been traditionally performed by using dual-energy-X-ray-absorptiometry (DXA) or quantitative computed tomography (QCT) assessing bone mineral density (BMD) (1). BMD is used in clinical routine to determine osteoporosis-associated fracture risk. Osteoporosis is defined as a skeletal disorder characterized by compromised bone strength predisposing an individual to an increased risk for fractures (2). It is classified as a public health problem, since osteoporosis-related fractures are associated with a reduction in quality of life and an increased morbidity and mortality (3, 4). However, BMD accounts for only 60–70% of the variation in bone strength (5) and the BMD values of subjects with and without osteoporotic fracture overlap (6). Measurements of trabecular bone microstructure, based on high-resolution imaging techniques, in addition to BMD have shown to improve the prediction of the variation in bone strength (7–9).

Despite the focus on the mineralized bone component for osteoporosis diagnostics, recent studies have highlighted the potential role of the non-mineralized bone component in bone health (10–14). Bone marrow fills the cavities of trabecular bone and it primarily consists of adipocytes (yellow marrow regions) or adipocytes and hematopoietic red blood cells (red marrow regions). It is well known that osteoporosis is associated with an increased bone marrow fat mass due to a shift of differentiation of mesenchymal stem cells to adipocytes rather than to osteoblasts (11, 13, 15). Multiple studies have shown that higher bone marrow fat fraction (BMFF) values are associated with lower BMD values (12, 14, 16–26).

Bone marrow fat has a distinctly different function compared to other white and brown adipose tissue depots or ectopic fat depots in the human body (27) and might play a role in the pathophysiology of metabolic disorders. Metabolic diseases, including obesity and diabetes, are known to have a complex and still poorly understood relationship to bone health. There seems to be a higher fracture-related morbidity in obese than in non-obese women (28), and more fractures in diabetic than in healthy subjects (29–32). In addition, there is a growing interest to study the relationship between bone marrow fat mass and fat in other depots regarding obesity and type 2 diabetes mellitus (T2DM).

In this context, imaging biomarkers are emerging to non-invasively study the properties of the non-mineralized component of bone (33). Dual-energy CT (DECT) allows to simultaneously determine bone marrow fat and BMD (34). In contrast to DECT, magnetic resonance (MR) allows the quantitative assessment of bone marrow without radiation exposure. MR enables reliable measurements of bone marrow water-fat and fatty acid composition with different methods including magnetic resonance imaging (MRI) and magnetic resonance spectroscopy (MRS) (35, 36).

The purpose of the present work is to review the currently available literature on MR-based assessment of bone marrow fat in the context of osteoporosis, T2DM, and obesity.

## MR-Based Assessment of Bone Marrow Fat

### Literature Research

Electronic searches in PubMed (http://www.ncbi.nlm.nih.gov/pubmed) were performed up to March 2015 to identify relevant studies for this review. No starting date was entered for the electronic search to obtain the entire literature available in PubMed. Search terms used included “Bone Marrow,” “Bone Marrow Fat,” “Bone Marrow Adipose Tissue,” “Magnetic Resonance Imaging,” “Magnetic Resonance Spectroscopy,” “Osteoporosis,” “Obesity,” and “Diabetes.” The search was restricted to studies in humans. The reference lists of relevant articles were also screened.

### MR Methods

T1-weighted imaging (T1WI), MRS, and chemical shift encoding-based water–fat imaging have been previously used to assess BMFF.

#### T1-Weighted Imaging

T1-weighted imaging is not technically demanding and has been mostly applied on the pelvis, hip, and spine. A recent study has even introduced a score analog to the DEXA *t*-score based on the mean signal-to-noise ratio of the L1–L4 vertebral bodies in T1WI (37). Measurements of bone marrow fat volume have been also proposed by applying thresholds on T1WI to extract bone marrow fat voxels (25). The applied threshold was usually set at the same gray-scale level as subcutaneous adipose tissue. The intra- and interobserver reproducibility for the assessment of bone marrow fat volume in T1WI images expressed as coefficient of variation (CV) amounted 0.9% (intraobserver) and 2.2% (interobserver) (for the post-processing) (38). The main error source for the calculation of bone marrow fat volume based on T1WI results from partial volume effects and threshold selection, especially in regions with red marrow.

#### Magnetic Resonance Spectroscopy

**Figure 1:**
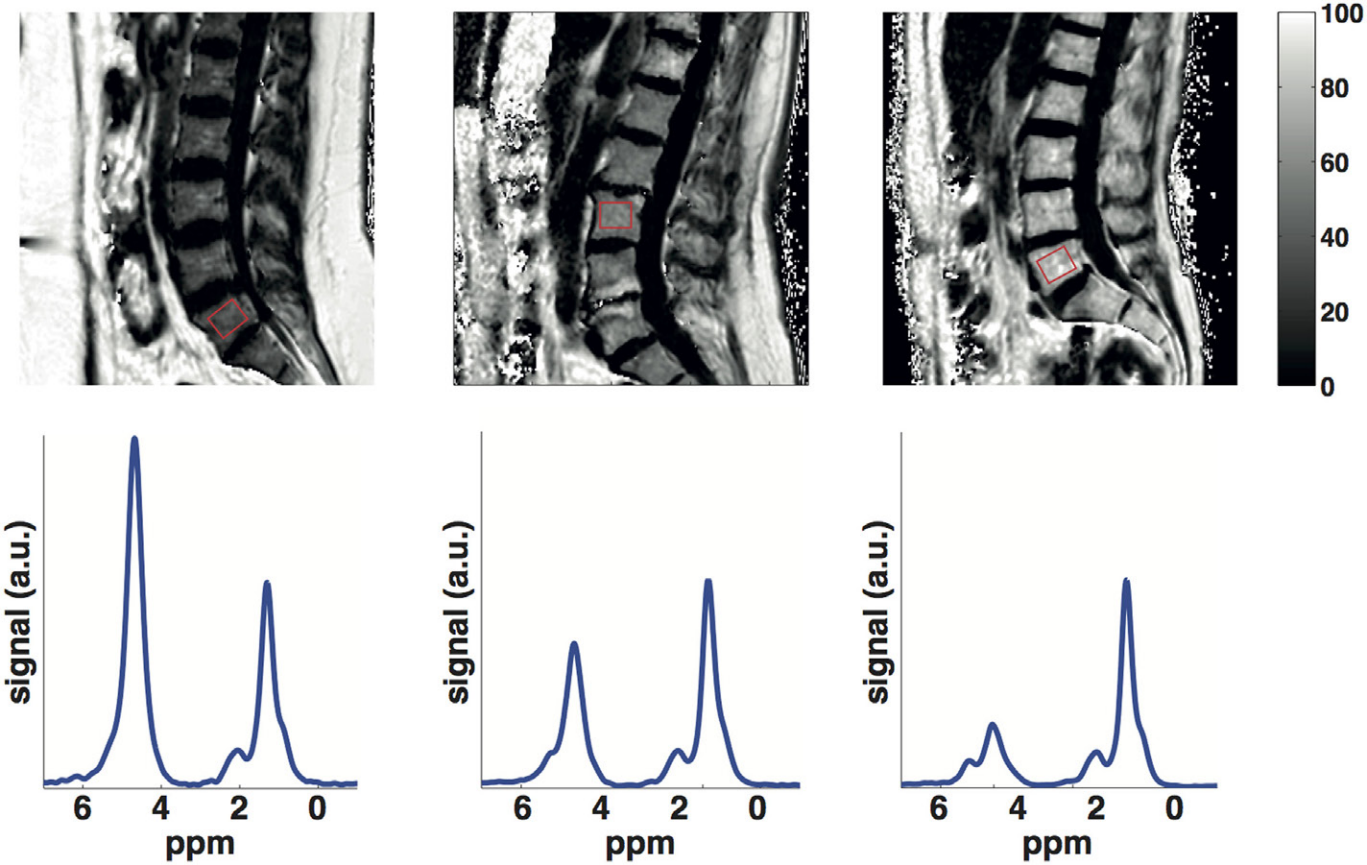
**Lumbar vertebral bone marrow PDFF maps (first row) and single-voxel MR spectra (second row) in a young subject (25 years), an old subject (60 years) with normal BMD (spine t-score = 3.7) and an old subject (60 years) with osteoporosis (spine *t*-score = −3)**. Bone marrow PDFF increases with age and lower BMD: the young subject had a PDFF on the L5 vertebral body (red box) equal to 32.4%, the old subject with normal BMD had a PDFF on the L3 vertebral body (red box) equal to 48.9% and the old subject with osteoporosis had a PDFF on the L5 vertebral body (red box) equal to 67.5%.

Proton-MRS (1H-MRS) is considered as the MR gold standard for bone marrow fat quantification (Figure 1). Point-resolved spectroscopy (PRESS) and stimulated echo acquisition mode (STEAM) single-voxel 1H-MRS sequences have been most commonly used for the characterization of the fat spectrum in the bone marrow at the pelvis, spine, and hip. Based on MR spectra, fat fraction and fatty acid composition parameters can be determined (36). The average CV of vertebral BMFF was reported to be 1.7% (on the same day with repositioning). Griffith et al. recruited 36 subjects who underwent MRS in the femoral neck and head, as well as in the subtrochanteric region of the femoral shaft to measure bone marrow fat content (39). They found the best reproducibility (between two measurements within 1 week) in the femoral head (interclass correlation 0.85), followed by the femoral shaft (interclass correlation 0.83), and the femoral neck (interclass correlation 0.78). Another recent study that examined reproducibility of 1H-MRS vertebral BMFF found a CV of 9.9% (SD 0.08) for a 6-week reproducibility and a CV of 12.3% (SD 0.10) for a 6-month reproducibility (40).

However, most of the above studies did not account for the difference in T2 relaxation times between the water- and fat components and measured a signal-weighted fat fraction at a single echo time (TE), dependent on the employed TE value and field strength. A multi-TE MRS measurement combined with T2 estimation routines instead removes the confounding effects of water–fat T2 differences and results in the proton density fat fraction (PDFF), which is independent of the employed experimental settings (41).

In addition, bone marrow spectra are characterized by strongly overlapping water and fat peaks due to susceptibility effects from the trabecular bone (41, 42): water and olefinic fat peaks overlap significantly, especially in the spine. The *a priori* knowledge of the chemical structure of triglycerides can be employed to improve the robustness of water peak extraction by constraining the area of the olefinic fat peak to the main fat peaks (41–43). However, the quantification of fat unsaturation relies on the extraction of the olefinic fat peak on an individual spectrum basis and remains a major technical challenge in the presence of a strong water peak (i.e., in the spine). Previous studies measuring vertebral bone marrow fat unsaturation based on short-TE spectra have reported moderate reproducibility values (same day, with repositioning) (36). Increasing the TE (44) or adding diffusion-weighting (45, 46) have been recently proposed to reduce the water peak height and more robustly extract the olefinic peak in vertebral bone marrow spectra.

#### Water–Fat Imaging

In contrast to single-voxel MRS, chemical shift encoding-based water–fat imaging allows the spatially resolved assessment of the BMFF (Figure 1). High-resolution BMFF mapping is highly advantageous due to the heterogeneous distribution of bone marrow in most regions (e.g., proximal femur, spine). However, several confounding factors have to be considered when measuring bone marrow PDFF by using water–fat imaging, including the presence of multiple peaks in the fat spectrum (47, 48), T1-bias (49, 50) and T2*-decay effects (47, 51). The presence of trabecular bone most importantly shortens the T2* of water and fat components, inducing a rapid decay of the measured gradient echo signal with echo time (42, 52). After correcting for T2* decay effects, a good agreement was reported *in vivo* between MRS-based and imaging-based PDFF in both the proximal femur (42) and spine (52).

An *ex vivo* study was performed in trabecular bone specimens filled with water–fat emulsions of different fat fractions, showing an excellent agreement between imaging-based PDFF and the known PDFF of the emulsions (53). To validate the results of bone marrow water–fat imaging with a non-MRI reference, Arentsen et al. (54) and MacEwan et al. (55) performed studies comparing water–fat imaging with histological examinations. Water–fat imaging in both studies resulted in vertebral BMFF and water fraction values, which showed good correlation (*r* = 0.76–0.77) with the histological results.

As water–fat imaging methodologies become more broadly available, studies have been recently performed to evaluate the significance of a spatially resolved fat fraction map (56–60). In a recent study, Baum et al. investigated whole body spine water–fat imaging on 28 healthy subjects (56). They found an increased BMFF from the cervical to the lumbar level. Absolute precision error amounted to 1.7% on the average.

Despite a considerable amount of literature using MR methods to assess bone marrow properties in the pelvis and the long bones, vertebral bone marrow remains the region most extensively investigated in bone marrow studies of aging, osteoporosis, diabetes, and obesity (Table 1).

**Table 1:** **Summary of MR-based studies investigating vertebral bone marrow fat properties in osteoporosis, diabetes, and obesity**.

Study	Subjects	MR technique and bone marrow fat parameters	Main results for bone marrow fat
**Osteoporosis**			
Yeung et al. (26)	53 women	MRS for fat fraction and unsaturation	Higher fat fraction and lower fat unsaturation in osteoporotic than osteopenic and normal subjects
Griffith et al. (19)	90 men	MRS for fat fraction	Higher fat fraction in osteoporotic than osteopenic and normal subjects
Griffith et al. (20)	103 women	MRS for fat fraction	Higher fat fraction in osteoporotic than osteopenic and normal subjects
Patsch et al. (66)	69 women	MRS for fat fraction and unsaturation	Fat unsaturation negatively associated with prevalence of fragility fractures
Kühn et al. (57)	51 subjects	Water-fat imaging for PDFF	Higher PDFF in osteoporotic than normal subjects
Karampinos et al. (21)	10 specimens	MRS for fat fraction (*ex vivo*)	Fat fraction negatively associated with failure load
**Diabetes and obesity**			
Bredella et al. (70)	47 women	MRS for fat fraction	Fat fraction positively associated with visceral fat volume
Baum et al. (16)	26 women	MRS for fat fraction and unsaturation	Fat unsaturation lower in diabetics compared to non-diabetics
Bredella et al. (67)	35 men	MRS for fat fraction	Fat fraction negatively associated with bone strength parameters
Bredella et al. (68)	106 women	MRS for fat fraction	Fat fraction positively associated with intra-hepatic and intramyocellar lipids
Bredella et al. (69)	79 women	MRS for fat fraction	Fat fraction increased after growth hormone therapy
Cordes at al. (72)	20 women	MRS for fat fraction	Fat fraction unchanged after a 4-week calorie restriction in obesity, but fat fraction changes associated with subcutaneous fat volume before intervention
Schafer et al. (22)	11 women	MRS for fat fraction	Fat fraction decreased only in diabetics after a gastric bypass surgery

### Bone Marrow Fat in Aging

The BMFF is well known to increase with age (Figure 1). The exact age dependence has been shown to differ between male and female subjects (61–63). A recent study has also emphasized the importance of T2-correction when using MRS to investigate the age dependence of BMFF (41). However, less is known about the age dependence of bone marrow fat unsaturation. Huovinen et al. recently investigated bone marrow fat unsaturation in young adults (15–27 years) (64). Thirty-five young adults from 27 to 35 years were included in their study, and they showed an increase of bone marrow fat unsaturation with age. The results suggest an association of bone marrow fat unsaturation with age in early adulthood and may represent the normal maturation of bone marrow.

### Bone Marrow Fat in Osteoporosis

#### Bone Marrow Fat Fraction

The MR-based vertebral bone marrow fat parameter studied most frequently in the context of osteoporosis is the fat fraction. Representative data from the authors own work, using a methodology similar to Baum et al. (65) and highlighting the effect of age and osteoporosis on bone-marrow fat fraction, are shown in Figure 1.

Most previous studies evaluating the relationship between BMD and BMFF have been based on single-voxel MRS. Griffith et al. performed a study in 103 female subjects to investigate the association between bone marrow fat and osteoporosis *in vivo* (20). They acquired DXA to stratify the group into healthy (*n* = 18), osteopenic (*n* = 30), and osteoporotic subjects (*n* = 55). MRS was performed at L3. The vertebral marrow fat fraction was significantly increased in osteoporotic subjects (67.8 ± 8.5%) compared with healthy subjects (59.2 ± 10.0%). Similar results were found in men. In another study, 90 men (42 subjects with normal BMD, 23 osteopenic, and 17 osteoporotic subjects) were recruited and obtained a measurement of the bone marrow fat content in L3 using MRS (19). The vertebral marrow fat fraction was significantly increased in subjects with osteoporosis (58.2 ± 7.8%) and osteopenia (55.7 ± 10.2%) compared to subjects with normal BMD (50.5 ± 8.7%).

Karampinos et al. recently examined ten vertebrae from human cadavers using MRS to assess BMFF, multi-detector computed tomography (MDCT) to determine BMD and trabecular bone microstructure parameters, and biomechanical testing to assess vertebral bone strength (21). They reported significant correlations between the MRS-based fat fraction and MDCT-based parameters (up to *r* = −0.72), and MRS-based fat fraction and vertebral failure load (*r* = −0.77). Thus, this study demonstrated that bone marrow fat volume is negatively associated with both bone microstructure and bone strength. However, further studies with larger number of specimens are needed in order to investigate whether the BMFF has an effect on bone strength after correcting for the contribution of BMD.

Kühn et al. performed one of the first bone marrow high-resolution fat fraction mapping studies in patients with osteoporosis. Their study included 51 patients who underwent DXA as well as water-fat imaging of the lumbar spine (57). According to the DXA results, the participants were divided into three subgroups: 92 healthy, 47 osteopenic, and 34 osteoporotic. The obtained PDFF was greater in osteoporotic than healthy vertebrae (62.4 ± 11.0% versus 56.3 ± 14.8%). Thus, water–fat imaging could be an alternative to the most commonly used MRS to assess bone marrow fat in the context of osteoporosis.

#### Bone Marrow Fat Unsaturation

The second MR-based bone marrow fat parameter, which has been linked to bone matrix loss, is bone marrow fat unsaturation. Yeung et al. analyzed bone marrow fat composition in the context of osteoporosis (26). They included 53 women over 60 years of age and 12 young controls in their study. The subjects underwent DXA and MRS of the lumbar spine. Interestingly, the fat unsaturation level was significantly decreased in osteoporotic (0.091 ± 0.013) and osteopenic (0.097 ± 0.014) subjects compared to healthy subjects (0.114 ± 0.016) and young controls (0.127 ± 0.031). Patsch et al. investigated the vertebral bone marrow fat composition in diabetic and non-diabetic postmenopausal women with and without fragility fractures (66). In consistency with Yeung et al., the prevalence of fragility fractures was associated with −1.7% lower unsaturation levels and +2.9% higher saturation levels.

### Bone Marrow Fat in T2DM

Baum et al. performed a study on 26 subjects, including 13 non-diabetic and 13 treated diabetic women (16). The subjects underwent blood parameter analysis including plasma glucose and HbA1c. Images from mid T12 to mid L4 were acquired using MDCT to obtain SAT (subcutaneous adipose tissue), VAT (visceral adipose tissue), and TAT (total adipose tissue). MRS was obtained at L1, L2, and L3 to assess BMFF and unsaturation. The mean vertebral BMFF was similar in the diabetic women and the healthy controls (69.3 ± 7.5% versus 67.5 ± 6.1%; *P* > 0.05). However, the mean unsaturation level was significantly lower in the diabetic group (6.7 ± 1.0% versus 7.9 ± 1.6%; *P* < 0.05). Adjusted SAT and TAT correlated significantly with mean vertebral bone marrow fat content in the whole study population (*r* = 0.538 and *r* = 0.466; *P* < 0.05). Interestingly, significant correlations of mean vertebral bone marrow fat content with adjusted VAT and HbA1c were observed only in the diabetic group (*r* = 0.642 and *r* = 0.825; *P* < 0.05).

Similar results to the study by Baum et al. (16) were reported by Patsch et al. (66): diabetes was associated with a −1.3% lower unsaturation and +3.3% higher saturation levels. Diabetics with fractures showed the lowest marrow unsaturation and highest saturation. Therefore, the authors suggested that vertebral bone marrow fat unsaturation beyond fat fraction might have a potential for assessing the BMD-independent fracture risk.

### Bone Marrow Fat in Obesity

Bone marrow fat fraction has been shown to be a negative predictor of bone microarchitecture and mechanical properties in obese men (67). BMFF has been also shown to be positively associated with ectopic and serum lipid levels in obese men and women (68) and to increase after a 6-month growth hormone administration in obese women (69).

To get further insight into the relationship between bone marrow fat and obesity, Bredella et al. performed a study in 47 pre-menopausal women (70). The authors measured IGF-1 and growth hormone blood levels, and performed MRS at L4 and MDCT scans at the level of L4 for VAT determination. The vertebral BMFF was positively associated with VAT and inversely associated with IGF-1. The authors concluded that visceral fat might have detrimental effects on bone health, which may be mediated in part by IGF-1 as an important regulator of the fat and bone lineage. Wongdee et al. concluded in a recent review that obesity and insulin resistance because of hyperglycemia in T2DM may induce osteoblast and osteoclast dysfunction with a resulting lower bone turnover (71). However, the underlying cellular and molecular mechanisms of insulin resistance in osteoblasts, osteoclasts, and osteocytes remain unknown and require further investigation.

Cordes et al. examined a group of twenty obese women who underwent a 4 week calorie restriction of 800 kcal/day (72). They performed MRS of the bone marrow and liver. Furthermore, they determined blood fat values and acquired two-point Dixon images of the abdomen to measure SAT and VAT. Despite a significant reduction of the body mass index (BMI), SAT, VAT, triglycerides, and LDL (low density lipoproteins)-cholesterol, they observed no significant reduction of the vertebral BMFF. However, absolute BMFF changes were positively associated with SAT volume (*r* = 0.489) and negatively associated with non-adipose tissue volume (*r* = −0.493) before dietary intervention (67).

Schafer et al. examined the changes of the BMFF and bone mass after gastric bypass surgery (22). They included eleven women (six diabetic, five non-diabetic) who underwent bariatric surgery (Roux-y-gastric-bypass) and underwent L3/L4 MRS, anthropometric measurements, whole body fat, and BMD measurements. VAT was determined on a single slice at the level of L4 using MDCT. In consistency with previous studies, they reported a positive correlation between age and bone marrow fat content. Interestingly, mean bone marrow fat decreased (−7.5%, *p* = 0.05) in the diabetic subjects, while the non-diabetic women showed only a small change (+0.9%, *p* = 0.84). Despite the small sample size, this study highlighted that bone marrow fat behaves differently compared to other fat depots in patients without diabetes after gastric bypass surgery.

## Conclusion and Perspectives

In summary, MR-based assessment of bone marrow fat provides interesting insights into the pathophysiology of osteoporosis, T2DM, and obesity as well as the association of bone and metabolic disturbances. Currently available MR methods including MRS and water–fat imaging enable the non-invasive extraction of the BMFF and unsaturation. Very little is known about the underlying mechanisms. Furthermore, new research questions are evolving on the role of bone marrow adipocytes in bone remodeling, hematopoietic stem cell differentiation, and whole body homeostasis (73, 74), on novel non-invasive MR biomarkers specific to the distribution, composition, microstructure, and function of bone marrow adipocytes requiring further investigations.

## Author Contributions

Collection of literature: CC, TB, and DK. Development of technical parts: MD, SR, MND, and DK. Development of clinical parts: CC, TB, HH, JK, and DK. Manuscript preparation/revision: CC, TB, MD, SR, MND, HH, JK, and DK.

## Conflict of Interest Statement

DK receives Grant Support from Phillips Healthcare. The remaining authors declare that the research was conducted in the absence of any commercial or financial relationships that could be construed as a potential conflict of interest.
